# The Indirect or Model Method for Inlays and Crowns

**Published:** 1916-08

**Authors:** Edward B. Spalding

**Affiliations:** Detroit


### THE INDIRECT OR MODEL METHOD FOR INLAYS AND CROWNS.

BY EDWARD B. SPALDING, D.D.S., DETROIT.

The term "indirect method" probably prejudices many dentists from attempting to learn the impression and model method.

It suggests something lacking in accuracy, or else it seems too complicated or too time-consuming to deserve attention.

Instead of lacking accuracy, we feel it makes for better work, and when the making of correct models is learned, more and better inlay work will be done than by the direct method. This has been proven to the satisfaction of many successful men.

Dr. F. T. Van Woert prophesied some three years ago that "unless some reliable and practicable method for the production of cemented fillings is secured it will suffer the fate of many other valuable discoveries and become a thing of the past." We believe the indirect method will be the saving of the inlay method to many of the profession at least.

It is not that the inlay lacks merit but the technic of producing accurate work when done on the tooth itself in the mouth is too exacting for the skill and patience of the average dentist. If, however, one attempts the impression method without first becoming acquainted with the principles of it, he is very likely to meet with failure and condemn the whole process. The principles are not obscure nor difficult to learn, but are more easily shown in a clinic than described.

Those who have adopted the impression and model method are unanimous in claiming that it has been the means of improving their cavity preparation to a very great degree. It is difficult to be sure of cavity proportion, the lines in transparent enamel and the angles obscured in the shadows of a cavity in a natural tooth in position in the mouth, even when we have a definite form in mind. A correct model of a cavity shows us faults which we did not believe existed. In that way one soon learns to check up on his weak points and to study for better mechanical retention.

There are many advantages in the indirect method to both the operator and the patient.

The advantages to the operator are:

*First:* Shortening of operating time on the patient. We all know that the longer the operation on a single patient the greater the strain to both patient and operator.

Two patients for an hour each are not as fatiguing as one patient for two hours provided that the work is equally exacting.

*Second:* Comparative ease in obtaining a good impression of the cavity as against the more difficult fitting and carving of wax patterns or the burnishing of a matrix in the mouth.

*Third:* Better opportunity for occlusal and other anatomical restorations. It stands to reason that more exact and careful carvings can be done on a model which can be readily handled and observed than can be done on a tooth in the mouth.

Carving to restore sulci, marginal ridges, cusps and buccal and lingual contour are being deemed more and more important in tooth restoration, no matter what the material used. These anatomical restorations, when done in the mouth, require expert technic, and that is acquired by but few.

The articulated model in the laboratory makes this carving possible and comparatively easy to the average dentist or laboratory assistant.

*Fourth:* Not only the possibilities but the opportunity after the impression is taken to delegate the whole process of making the model and then the inlay or crown to a competent laboratory assistant, thereby relieving the operator of much laborious work which lessens to an extent the cunning of his fingers for operating. I am free to admit that a clever laboratory assistant who is devoting her time to the burnishing of matrices and baking of porcelain inlays and crowns, and the carving of wax forms for cast work, can outstrip me in the same work when I have divided my time between that work and operating at the chair.

*Fifth:* When an office is equipped for the indirect method more and better work can be done each day than by the direct method.

*Sixth:* Ease in finishing and polishing gold inlays when done on a model.

*Seventh:* Much better margins and fit for porcelain inlays and porcelain jacket crowns.

*Eighth:* The opportunity to make corrections in a piece of work on the model, and if the piece is a failure, as sometimes happens, the model is a willing patient to submit to reconstruction.

*Ninth:* Operations which are difficult or even impossible by the direct method are comparatively simple by the impression and model method.

To the patient the advantages are chiefly two, viz.:

*First:* The lessened time for operating directly in his mouth. There is no patient who chooses to have more work done in his mouth than is necessary.

The indirect method does lessen the time for the patient. After having work done by the indirect method a patient is very loath to submit to the direct method.

*Second:* It also eliminates much disagreeable work. It is much more agreeable for the patient to have an impression of an extensive cavity running beneath the gum margin taken with a properly fitted matrix and modeling compound than to have a wax pattern trimmed and finished along this hidden cavity margin.

Also the finishing of a gold inlay on the tooth in the mouth is a most disagreeable procedure for a patient, and when he finds there is a way to avoid it, he is going to do so.

Today the public is aroused as never before to the necessity of caring for the teeth; consequently the demand for the services of the dentist is greater and there is being done more of all kinds of dentistry.

Discriminating people are on the alert to find better, more skillful, more ''humanitarian'' dentistry as they submit themselves for operations which a few years ago were considered unnecessary.

The operator who studies to improve his technique for the consideration of his patients soon finds his time filled to overflowing.

The indirect method is a great aid to humanitarian as well as skillful dentistry.—*Dental Summary.*

